# Lattice Rotation and Deformation Mechanisms under Tensile Loading in a Single-Crystal Superalloy with [001] Misorientation

**DOI:** 10.3390/ma17061368

**Published:** 2024-03-16

**Authors:** Xiangyu Gao, Zheng Zhang, Liyu Liu, Chunhu Tao

**Affiliations:** 1 School of Materials Science and Engineering, Beihang University (BUAA), Beijing 100191, China; 2 AECC Beijing Institute of Aeronautical Materials, Beijing 100095, China; liuliyu@aliyun.com (L.L.); taochunhu1208@sina.com (C.T.); 3 AVIC Failure Analysis Center, Beijing 100095, China; 4 Key Laboratory of Aeronautical Materials Testing and Evaluation, Aero Engine Corporation of China, Beijing 100095, China; 5 Beijing Key Laboratory of Aeronautical Materials Testing and Evaluation, Beijing 100095, China

**Keywords:** single-crystal superalloy, misorientation, deformation mechanism, lattice rotation

## Abstract

This study investigates how deviation angles close to the [001] orientation affect the tensile properties and deformation behavior of a nickel-based single-crystal superalloy at room temperature. The research focuses on samples with deviation angles of 3°, 8°, and 13° from the [001] orientation and examines their strength and ductility. We employed scanning electron microscopy (SEM), electron backscatter diffraction (EBSD), and transmission electron microscopy (TEM) to explore the deformation micro-mechanisms at varying angles. Findings reveal that strength decreases and ductility increases as the deviation angle widens within the [001] vicinity. The study emphasizes that <110> octahedral slip-driven crystal slip and rotation are crucial for understanding tensile deformation. The deformation differences in samples at varying angles are attributed to the differential engagement of mechanisms. Specifically, at lower angles, reduced ductility and increased strength are due to short lattice rotation paths and work hardening causing superlattice stacking faults (SSFs) to slip in two directions on the {111} plane within the γ′ phase. As the angles increase, the lattice rotation paths extend, and Shockley partial dislocations (a/6<112>) accumulate in γ channels. This process, involving SSFs moving in a single direction within the γ′ phase, results in higher ductility and reduced strength.

## 1. Introduction

The thrust-to-weight ratio serves as a key metric for evaluating the advancement of aerospace engines, where improvements in this ratio necessitate increased turbine inlet temperatures, thereby increasing the thermal endurance requirements for the hot section components, including turbine blades and disks. High-pressure turbine rotor blades, which are located in the hot section of the engine, are subjected to the most adverse conditions, including high temperatures, centrifugal forces, aerodynamic loads, and vibrations [1,2]. Currently, nickel-based single-crystal superalloys, cast using directional solidification techniques such as the selector or seed crystal method, are the predominant material used for high-pressure turbine rotor blades. This process eliminates grain boundaries that could potentially initiate cracking, resulting in single-crystal blades. Compared to equiaxed crystal cast superalloys, this significantly increases their thermal strength and resistance to thermal corrosion [3,4,5]. In addition, high-pressure turbine blades made from single-crystal superalloys are equipped with complex cooling systems and thermal barrier coatings to further enhance their high-temperature resistance [6,7,8,9].

Anisotropy distinguishes nickel-based single-crystal superalloys from polycrystalline materials, with extensive research confirming differences in microstructural morphology and mechanical properties among the [001], [011], and [111] orientations [10,11,12,13,14,15,16]. The [001] orientation, which has a lower modulus of elasticity, is the preferred orientation for single-crystal blades in engineering applications. However, during the actual casting process of the blades, due to factors such as alloy properties, processing conditions, and casting structure, it is difficult to ensure that the principal stress axis of the blade is perfectly aligned with the [001] orientation of the single crystal, often resulting in deviations. As turbine inlet temperatures increase and single-crystal superalloys evolve, the incorporation of more refractory elements characterized by low diffusion coefficients complicates the control of the crystal orientation of single-crystal blades [4,17,18]. In practical engineering applications, blades that deviate from the [001] orientation by up to 15° are generally considered acceptable, reflecting a compromise between casting difficulties and performance requirements. In actual service, blades with varying degrees of misalignment inevitably introduce variability into engine performance, and there is increasing evidence that larger misalignment angles degrade the tensile, creep, and fatigue properties of the blade [19,20,21,22].

Crystal slip represents the principal deformation mechanism in single-crystal superalloys, with slip-system activation governed by Schmid’s law. However, the anisotropy of single-crystal superalloys cannot be simply represented by the resolved shear stress of a single-slip system; to do so would reduce the anisotropy to a simple function of the Schmid factor, which is defined entirely by crystallographic relationships. MacKay’s research [23] demonstrates that lattice rotation occurs in single-crystal superalloys during stress creep deformation. Slip systems are activated only after the lattice has rotated to a particular orientation. The [011] orientation requires significant rotation to facilitate cross-slip, resulting in considerable primary creep strain and the shortest creep life for crystals in this orientation. Subsequent studies on lattice rotation have emerged [24,25,26], primarily addressing the creep deformation of single-crystal superalloys under asymmetric loading. Asymmetric loading is characterized by a certain angular deviation of the specimen’s orientation from the [001] direction, leading to a misalignment between the crystallographic axes and the applied loading axes. This misalignment results in asymmetric loading on the crystal axis during the experimental procedures. These studies investigated the activation of slip systems and the conditions for lattice rotation based on Schmid’s law. However, the deformation mechanism of single-crystal superalloys exhibits a significant correlation with temperature, demonstrating a clear distinction between deformation behaviors at room temperature and at elevated temperatures [27,28]. The behavior of lattice rotation is distinctly specific, highlighting several issues that require resolution. These encompass the conditions for initiating lattice rotation during room temperature tensile deformation of single-crystal superalloys, the paths of such rotation, and the micro-mechanisms of dislocation motion associated with lattice rotation.

When single-crystal superalloys are used for turbine blades in aviation engines, those blades with a principal stress axis deviating from the [001] direction by up to 15° are considered acceptable. This indicates that, within the same engine, blades with deviation angles ranging from 0 to 15° may be used simultaneously. The difference in deviation angles results in variability in performance among the blades, also reducing the operational stability of the engine. As is well known, even a single blade’s failure can have catastrophic effects on the engine. To enhance the safety and reliability of engine operation, this study investigates the differences in performance and damage behavior caused by varying deviation angles within the engineering acceptance range. Ingots with deviations from the [001] orientation of 3°, 8°, and 13° were selected and mechanically machined into tensile specimens. The orientations of these specimens were remeasured using a single-crystal X-ray diffractometer (SC-XRD). Tensile tests were then performed at room temperature using an Instron 5887 tensile tester to provide data on ultimate tensile strength, yield strength, elongation, and cross-sectional shrinkage, among other strength and plasticity parameters. The fractography, microstructure, slip lines, micro-plastic deformation, and dislocation morphologies of the specimens post-tensile testing were analyzed and characterized using a scanning electron microscope system (SEM), electron backscatter diffraction (EBSD), and a transmission electron microscope (TEM). The relationship between mechanical properties and deformation mechanisms under different deviation angles was analyzed, providing data support for the mechanical damage behavior within the [001] orientation range of nickel-based single-crystal superalloys.

## 2. Experiment and Methods

### 2.1. Materials Preparation

**Figure 1:**
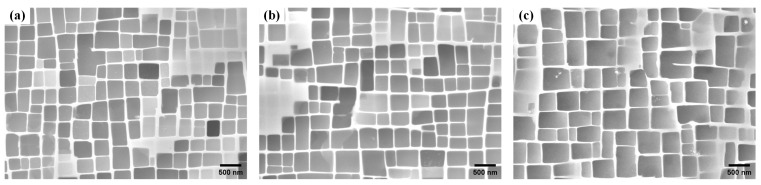
γ/γ′ two-phase structure of single-crystal superalloy cast ingots at (**a**) 3° deviation from [001]; (**b**) 8° deviation from [001]; (**c**) 13° deviation from [001].

The second-generation nickel-based single-crystal superalloy prepared by spiral crystal selection is used as the raw material, and the chemical composition is listed in Table 1. A stepwise solution heat treatment was applied: 1290 °C for 1 h, followed by 1300 °C for 2 h, then 1315 °C for 4 h, all followed by air cooling. Subsequently, a two-stage aging heat treatment was conducted: 1120 °C for 4 h, air cooled, then 870 °C for 32 h, air cooled. The two aging processes were aimed at obtaining uniformly dispersed γ′ precipitates and adjusting the cuboidal nature of the γ′ precipitates [3]. The deviation angles from the [001] orientation along the length of each ingot were measured using a Bruker D8 Venture type single-crystal X-ray diffractometer (SC-XRD, Karlsruhe, Germany) as 3°, 8°, and 13°, labeled as L#, M#, and H#, respectively. To prepare metallographic specimens by selectively sectioning ingot materials, the Struers CitoPress-30 (Ballerup, Denmark) metallographic preparation system was first utilized for embedding specimens. The embedding process was conducted at a temperature of 150 °C, a pressure of 30 MPa, and for a duration of 15 min. Subsequently, the specimens were polished using the Saikasi Alpha-351 (Guizhou, China) system, with a grinding rotation speed of 300 r/min. Successive grinding steps involved sandpapers of grit sizes 120, 400, 600, and 1200, followed by polishing at a rotation speed of 150 r/min, utilizing diamond suspension as the polishing fluid. The specimens were etched with a copper sulfate etchant (CuSO_4_: HCl: H_2_O = 20 g: 100 mL: 80 mL) for 3 s at room temperature and observed using a scanning electron microscope system (SEM, Zeiss Gemini SEM 500, Jena, Germany) to examine the γ/γ′ phase structures at different deviation angles, as shown in Figure 1.

### 2.2. Specimen Processing and Tensile Test

**Figure 2:**
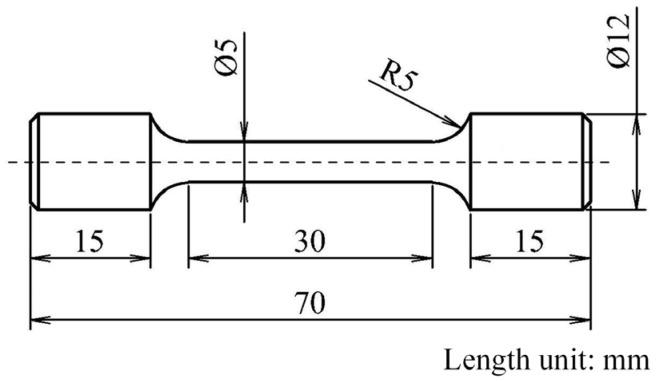
Schematic illustration with dimensions of single-crystal superalloy specimens processing for the tensile test.

Ingots with different deviation angles were processed into specimens as shown in Figure 2, ensuring that the longitudinal direction of the ingot was the axial direction of the specimen. Two specimens were manufactured from each ingot, and their orientations were remeasured using a single-crystal X-ray diffractometer (SC-XRD) to ensure that the orientation of the specimen was essentially unchanged from that of the ingot. Specimens from ingots with a 3° deviation were labeled as 1# and 2#, those with an 8° deviation were labeled as 3# and 4#, and those with a 13° deviation were labeled as 5# and 6#. The remeasured specimen orientations are shown in Table 2, and the results indicate that the deviation angle changes minimally after processing, which can represent the original deviation angle of the ingot. An Instron 5887 (Norwood, MA, USA) tensile testing machine was used to conduct tensile tests on the six specimens, following the Chinese aviation industry standard HB 5143-1996. The tests were performed in an environment of 23 °C, and the ultimate tensile strength (*σ_b_*), yield strength at 0.2% residual deformation (*σ_p_*_0.2_), elongation (*δ*), and cross-sectional shrinkage (*ψ*) were measured for each specimen.

### 2.3. Fracture Behavior Characterization

Using the mechanical polishing and etching methods described in Section 2.1, metallographic specimens of the near-fracture cross section were prepared. The fracture morphology, side-slip line features, and the microstructural state of the near-fracture specimens were observed and analyzed using a scanning electron microscope system (SEM, Zeiss Gemini SEM 500). Cross-section specimens near the fracture of samples with different deviation angles were prepared for electron backscatter diffraction (EBSD) analysis using mechanical polishing followed by vibratory polishing, consistent with the method described in Section 2.1. Vibratory polishing was conducted using a ATM Saphir Vibro (Mammelzen, Germany) polisher set to automatic mode, with a vibration frequency of 90 Hz, an intensity of 80%, and a duration of 6 h. Subsequently, an HKL Nordly Max3 (Oxford, UK) type EBSD was used to detect and analyze the orientation and plastic deformation patterns of the cross-sections of the samples. Transmission electron microscope (TEM) specimens near the fracture were prepared using mechanical thinning followed by electrolytic twin-jet polishing. Initially, specimens were mechanically ground to a thickness of 60 μm, then thinned using the electrolytic twin-jet method with an electrolyte solution of perchloric acid:alcohol = 1:9, at 30 V and −20 °C. Observations and analysis of dislocation configurations were subsequently carried out using an FEI Talos F200S type TEM (Valley City, ND, USA), with an observation acceleration voltage of 200 kV.

## 3. Results

### 3.1. Tensile Properties

**Figure 3:**
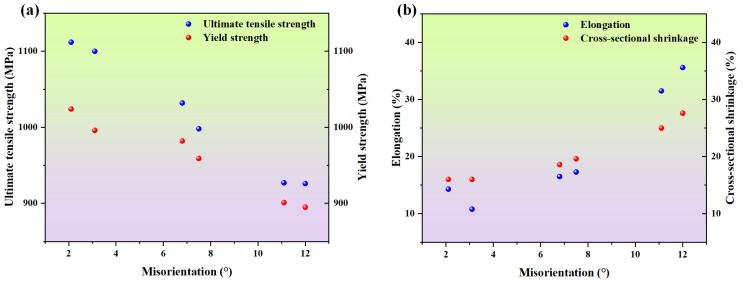
Tensile properties of single-crystal superalloy with different misorientation: (**a**) ultimate tensile strength (*σ_b_*) and yield strength (*σ_p_*_0.2_); (**b**) elongation (*δ*) and cross-sectional shrinkage (*ψ*).

Table 3 and Figure 3 illustrate the room temperature tensile properties of specimens with varying degrees of deviation from the [001] orientation axis, including ultimate tensile strength (*σ_b_*), yield strength (*σ_p_*_0.2_), elongation (*δ*_5_), and cross-sectional shrinkage (*ψ*). For samples with a deviation angle of less than 5°, 1# (3.1° deviation from [001]) and 2# (2.1° deviation from [001]), the ultimate tensile strengths were 1100 MPa and 1112 MPa, the yield strengths were 996 MPa and 1024 MPa, the elongations were 10.8% and 14.3%, and the cross-sectional shrinkages were both 16.0%, respectively. For samples with a deviation angle between 5° and 10°, 3# (7.5° deviation from [001]) and 4# (6.8° deviation from [001]), the ultimate tensile strengths were 998 MPa and 1032 MPa, the yield strengths were 959 MPa and 982 MPa, the elongations were 17.3% and 16.5%, and the cross-sectional shrinkage were 19.6% and 18.6%, respectively. For samples with the deviation angle between 10° and 15°, 5# (11.1° deviation from [001]) and 6# (12.0° deviation from [001]), the ultimate tensile strengths were 927 MPa and 926 MPa, the yield strengths were 911 MPa and 903 MPa, the elongations were 31.5% and 35.6%, and the cross-sectional shrinkages were 25.0% and 27.6%, respectively. It can be observed that as the deviation angle increases, both the ultimate tensile strength and yield strength show a decreasing trend, while the elongation and cross-sectional shrinkage exhibit an increasing trend. This demonstrates the characteristic decrease in strength and enhancement of ductility in single-crystal superalloys.

### 3.2. Fracture Behavior Observation

#### 3.2.1. Fracture Morphology

**Figure 4:**
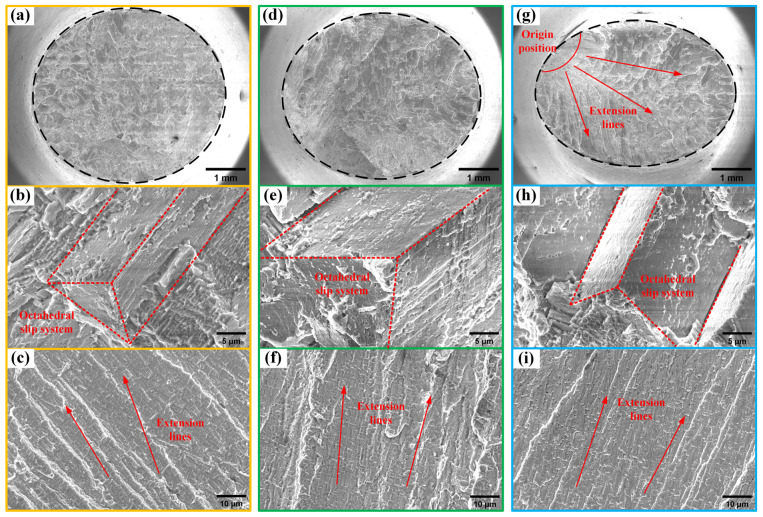
SEM morphology of fractures with different misorientations: overall fracture morphology, slip steps, extension lines of (**a**–**c**) L#—3° deviation from [001]; (**d**–**f**) M#—8° deviation from [001]; (**g**–**i**) H#—13° deviation from [001].

The fracture surfaces of samples with different angles of deviation from the [001] direction were examined using SEM in the secondary electron mode at a working distance of 10 mm and a voltage of 20 kV. The fracture characteristics of the two samples prepared from the same ingot were similar; hence, one sample fracture from each ingot was selected for description. This approach represents ingots L#, M#, and H# with deviation angles from the [001] direction of 3°, 8°, and 13°, respectively, and was adopted for subsequent analyses and characterizations. Figure 4 displays the fracture morphologies of the samples at different deviation angles. Macroscopically, the fracture cross section transitions from circular to elliptical as the deviation angle increases, indicating significant lattice rotation and plastic deformation in samples with larger deviation angles during the tensile test. Microscopically, the fracture surfaces of the three specimens with different deviation angles all exhibited quasi-cleavage fracture characteristics, composed of slip steps and tear ridges, with the slip steps showing typical octahedral slip features. The proportions of slip steps and tear ridges on the fracture surfaces of specimens with different deviation angles varied. For the specimen with a deviation angle of only 3° (L#), fewer tear ridges and more slip steps were observed, indicating that quasi-cleavage fractures occurred almost simultaneously at different locations on the fracture surface, forming a section perpendicular to the stress loading axis. As the deviation angle of the specimen increased, the number of tear ridges also increased. When the deviation angle reached 13.0° (H#), a clear tearing direction from one side to the other was visible across the entire fracture surface. At this point, quasi-cleavage fractures no longer occurred simultaneously at different locations on the fracture surface, but rather in a distinct sequence. This is due to the increased degree of asymmetric loading with increasing deviation angle, which, according to Schmid’s law, leads to different shear stresses required to activate slip systems at different locations on the fracture surface, causing a sequence in the activation of slip systems and, consequently, an overall tearing expansion phenomenon on the fracture surface.

#### 3.2.2. Slip System Features

**Figure 5:**
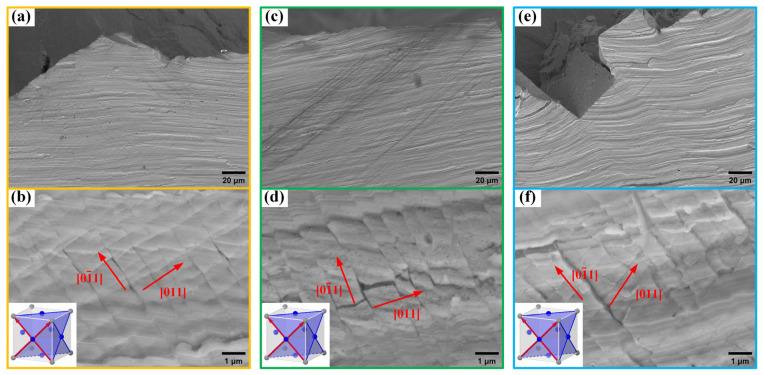
SEM morphology of slip lines on specimen surfaces with different misorientations: (**a**,**b**) L#—3° deviation from [001]; (**c**,**d**) M#—8° deviation from [001]; (**e**,**f**) H#—13° deviation from [001].

Surface slip lines near the fracture surfaces of samples with different deviation angles from the [001] direction were examined using SEM in the secondary electron mode, with a working distance of 10 mm and a voltage of 20 kV. Figure 5 illustrates the slip line morphologies on the fracture surfaces of the L#, M#, and H# oriented samples. All three samples showed two symmetrical slip directions in the <110>{111} octahedral slip system, including [011] and (0$\bar{1}$1). However, it was observed that for the L# orientation with only a 3° deviation, the activation of the two slip directions was more uniformly distributed, with good symmetry and no significant plastic deformation. In contrast, the M# orientation with an 8° deviation and the H# orientation with a 13° deviation both exhibited a predominance of one slip direction, accompanied by plastic deformation. This indicates that an increase in the deviation angle leads to an increase in the degree of asymmetric loading on the crystal axis. According to Schmid’s law, there is a sequence in the shear stress required to activate slip systems, resulting in the uneven activation of slip systems.

#### 3.2.3. γ/γ′ Morphology

**Figure 6:**
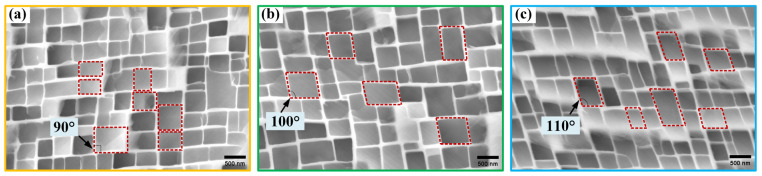
γ/γ′ phase torsional deformation after tensile test: (**a**) L#; (**b**) M#; (**c**) H# (3°, 8°, and 13° deviation from [001], respectively).

The cross-sectional microstructures near the fracture surfaces of samples with different deviation angles from the [001] direction were observed using SEM in the secondary electron mode, with a working distance of 10 mm and a voltage of 20 kV. Figure 6 displays the γ/γ′ phase morphologies near the fracture surfaces of the L#-, M#-, and H#-oriented samples with different deviation angles. The sample with an initial deviation angle of only 3° (L# orientation) retained its original cubic γ′ phase characteristics without noticeable torsional deformation after the tensile test. The sample with an initial deviation angle of 8° (M# orientation) exhibited torsional deformation of the γ′ phase, with a deformation angle of about 10°, while the sample with an initial deviation angle of 13° (H# orientation) showed even greater torsional deformation of the γ′ phase, about 20°. These observations correspond to the elliptical transformation of the cross-sections shown in Figure 4, indicating that the elliptical process of the cross-section is due to torsional deformation of the γ′ phase. This torsional deformation results from lattice rotation during asymmetric loading. The degree of lattice rotation increases with the increase in deviation angle, leading to a more pronounced degree of elliptical fracturing and distortion of the γ′ phase as the deviation angle increases.

### 3.3. EBSD Analysis

#### 3.3.1. Orientation Variation

**Figure 7:**
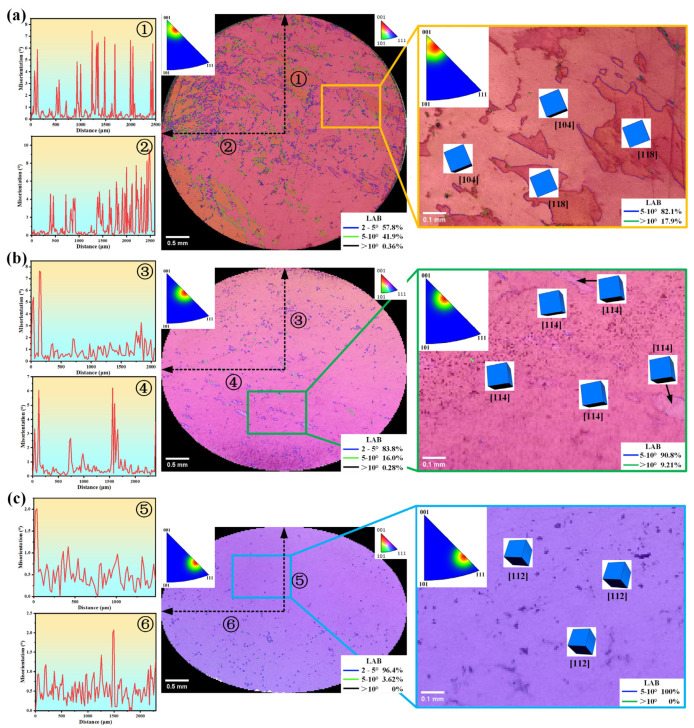
Low-angle boundaries (LABs) and orientation change analysis of the cross section near the fracture surface after the tensile test: (**a**) L#; (**b**) M#; (**c**) H# (3°, 8°, and 13° deviation from [001], respectively).

EBSD was used to examine the cross-sections near the fracture surfaces of samples with different deviation angles (L#, M#, and H# orientations) across the entire lateral interface, with a step size of 20 μm. AztecCrystal 2.1 software was utilized for statistical analysis of the results. Figure 7 presents the analysis results of the low-angle boundaries (LABs) and orientation changes generated after tensile deformation of each sample, characterizing the microplastic deformation of each sample. After the tensile testing, the L# orientation sample with a 3° deviation exhibited extensive microplastic deformation near the fracture cross-section, resulting in numerous LABs. There were orientation differences on either side of these boundaries: 57.8% of the LABs were within the 2–5° range, 41.9% were within the 5–10° range, and 0.36% exceeded 10°. Point-to-point orientation difference line scans were conducted from the center of the examined cross-section towards the upper and left edges. The maximum orientation difference reached approximately 10°. For the M# orientation sample with an 8° deviation after stretching, the LABs are more concentrated within a smaller angular range, with 83.8% within the 2–5° range, 16.0% within the 5–10° range, and 0.28% exceeding 10°. The same orientation difference line scans showed that only at specific local positions could the orientation difference reach approximately 8°, while at all other positions, the orientation difference was less than 3°. For the H# orientation sample with a 13° deviation after stretching, the LABs showed a further concentration within a smaller angular range, with 96.4% within the 2–5° range, 3.62% within the 5–10° range, and none exceeding 10°. The same orientation difference line scans indicated that the maximum orientation difference was approximately 2°.

Regarding orientation changes, after tensile deformation, the orientation of the L# sample concentrated around the [001]–[111] boundary, with the concentrated area deviating 9.8° from the [001] direction, close to the [118] orientation at 1.2°, and a minor distribution near the [001]–[101] boundary, close to the [104] direction at 5.9°. The orientation of the M# sample concentrated around the [001]–[111] boundary, with the concentrated area deviating 18.3° from the [001] direction, close to the [114] direction at 2.5°. The orientation of the H# sample concentrated around the [001]–[111] boundary, with the concentrated area deviating 39.0° from the [001] direction, close to the [112] direction at 3.9°. Therefore, it can be observed that after tensile deformation, the angle of deviation from the [001] direction increased for samples with different deviation angles, and all tended toward the [001]–[111] boundary.

#### 3.3.2. Dislocations Density

**Figure 8:**
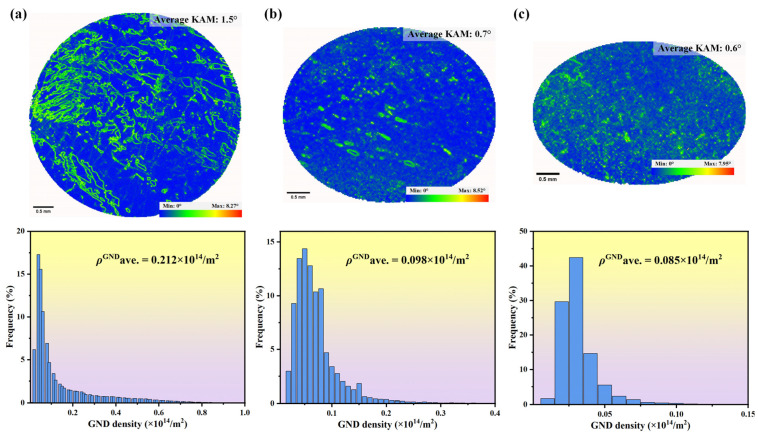
Kernel average misorientation and geometrically necessary dislocation analyses of the cross section near the fracture surface after the tensile test: (**a**) L#; (**b**) M#; (**c**) H# (3°, 8°, and 13° deviation from [001], respectively).

EBSD’s kernel average misorientation (KAM) and geometrically necessary dislocation (GND) analyses can be used to determine dislocation density. Figure 8 presents the KAM and GND analysis results for the entire cross-section near the fracture surfaces of samples oriented at different deviation angles (L#, M#, and H#). KAM reflects the degree of homogenization of the plastic deformation in the sample, with higher values indicating greater degrees of the plastic deformation or higher defect density. The average KAM values near the fracture cross-sections for the L#, M#, and H# orientations, with deviation angles of 3°, 8°, and 13°, are 1.5°, 0.7°, and 0.6°, respectively. This indicates that as the deviation angle increases, and the macroscopic elliptical extent of the fracture grows, the degree of local plastic deformation near the fracture decreases. Comparing Figure 7a and Figure 8a, it can be observed that the positions of higher KAM in the L# sample correspond to the locations of LABs. The theoretical GND can be calculated based on the KAM, as shown in Formula (1) [29]:

(1) $$\rho^{GND}=2{KAM}_{ave}/\mu b$$

In the formula, $\rho^{GND}$ represents geometrically necessary dislocations, indicating the extra storage of dislocations produced by plastic deformation of the sample. ${KAM}_{ave}$ is the average KAM value in the EBSD test area, *μ* is the step size chosen for the EBSD test, and *b* is the length of the Burgers vector, with the Burgers vector for nickel-based single-crystal superalloys is a/2<110>. The calculated GND densities for L#-, M#-, and H#-oriented samples after tensile deformation are 0.212 × 10^14^/m^2^, 0.098 × 10^14^/m^2^, and 0.085 × 10^14^/m^2^, respectively. This further indicates that as the angle of orientation deviation increases, the degree of local plastic deformation decreases.

### 3.4. Deformation Microstructure

Based on the analysis of microstructures and EBSD results, it can be determined that during the tensile testing process, samples with a deviation angle of 3° (L# orientation) exhibited micro-area plastic deformation, leading to the formation of LABs. The γ′ phase maintained its cubic structure well, with low degrees of lattice rotation, and the geometrically necessary dislocation density was significantly higher at the locations of LABs. For samples with deviation angles of 8° and 13° (M# and H# orientations), micro-area plastic deformation was not pronounced, but a noticeable torsional deformation of the γ′ phase occurred, indicating lattice rotation that increased with the deviation angle.

**Figure 9:**
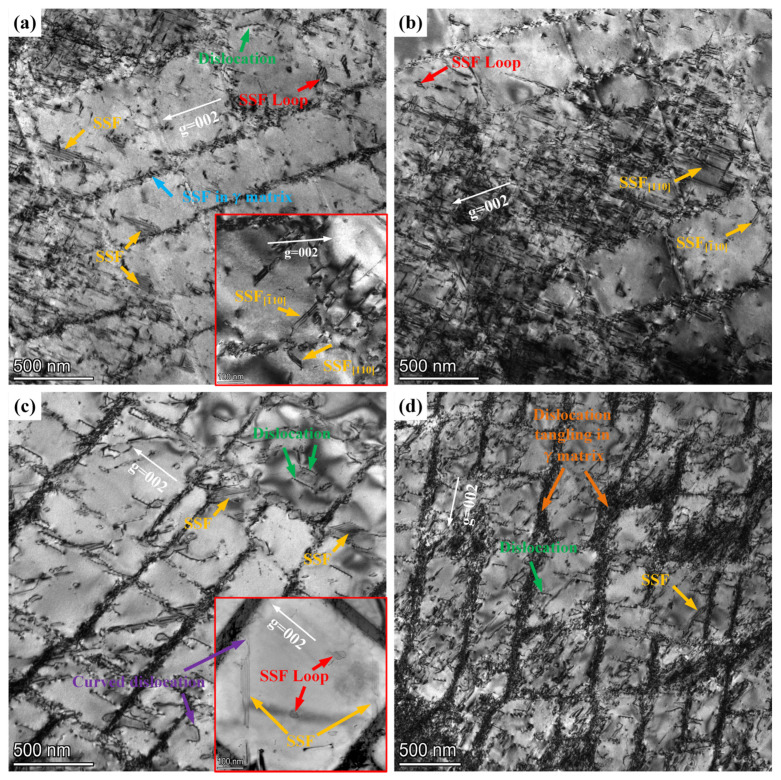
TEM dislocation analysis of the cross section near the fracture surface after the tensile test: (**a**,**b**) low and high dislocation density position of L#, respectively; (**c**) M#; (**d**) H# (3°, 8° and 13° deviation from [001], respectively).

TEM was used to observe and analyze the dislocation configurations in the cross-sections near the fracture surfaces of samples with different deviation angles (L#, M#, and H#). The analysis results, shown in Figure 9, under the operating vector g = 002, the samples with L# orientation exhibit significant differences in dislocation density at different locations (as shown in Figure 9a,b). The dislocation density within the γ channels is lower, predominantly characterized by the cutting of the γ′ phase by superlattice stacking faults (SSFs). In regions of varying dislocation density, SSFs in both the [110] and [$\bar{1}$10] directions are observed, as indicated by the yellow arrows in Figure 9a,b. This suggests that at least two slip systems were activated during the tensile process of the L# orientation. SSFs are present not only in the γ′ phase but also in the γ matrix, marked by blue arrows in Figure 9a, due to the addition of refractory elements like Re and Co, which results in a lower stacking fault energy (SFE) and is generated by interface dislocations. Furthermore, SSF loops are visible within the γ′ phase, marked by red arrows in Figure 9a,b, surrounded by super partial dislocations. Figure 9c illustrates the micro-dislocation morphology of samples with M# orientation, showing a twisting deformation from rectangular to parallelogram shapes in the γ′ phase. Compared to samples with L# orientation, there is a noticeable increase in dislocation density within the γ channels, and a reduction in SSFs within the γ′ phase (marked by yellow arrows in Figure 9c), predominantly in a single direction, indicating that tensile deformation is primarily governed by one slip system. In addition, incomplete dislocations are present in the γ′ phase (marked by green arrows in Figure 9c), along with curved dislocations (marked by purple arrows in Figure 9c). Figure 9d reveals the micro-dislocation morphology of samples with H# orientation, where the twisting deformation in the γ′ phase is more severe compared to L# and M# orientations, and a large number of tangled dislocations are visible within the γ channels (marked by orange arrows in Figure 9d), a result of lattice rotation. The density of dislocations cutting into the γ′ phase significantly increases, mainly comprising a large number of incomplete dislocations (marked by green arrows in Figure 9d) and a few SSFs (marked by yellow arrows in Figure 9d). These dislocations and stacking faults participate in the tensile deformation process of specimens with high deviation angles.

## 4. Discussion

Within the 15° range of near [001] orientation, samples with different deviation angles exhibited distinct strength and plastic deformation characteristics after stretching. Samples with smaller deviation angles (the L# sample deviating by 3°) showed high strength, low elongation, and low cross-sectional shrinkage, with a minor macroscopic elliptical extent of the fracture, and the fracture surface was essentially perpendicular to the specimen axis. Microscopically, there were numerous slip steps, and the distribution of octahedral slip lines was symmetrical, due to the small deviation angle causing minimal discrepancy between the crystal and loading axes, leading to symmetric activation of slip systems. The γ/γ′ phases largely maintained their initial form, indicating a minor degree of crystal rotation. Near the fracture surface, micro-area plastic deformation led to LABs, and the deformation mechanism was characterized by the cutting of the γ′ phase by SSFs, activating at least two slip systems. As the deviation angle increased (the M# sample deviating by 8° and the H# sample by 13°), strength decreased while elongation and cross-sectional shrinkage increased, along with a greater macroscopic elliptical fracturing and microscopic tearing. A sequence in the activation of octahedral slip lines emerged, due to the increasing deviation angle enlarging the discrepancy between the crystal and loading axes, increasing the degree of asymmetric loading and asymmetric activation of slip systems. The degree of twisting deformation in the γ/γ′ phases enhanced, indicating an increase in crystal rotation. Fewer LABs were produced after stretching, there was an increase in dislocation density within the γ channels microscopically, there was a decrease in the number of SSFs cutting into the γ′ phase, and primarily a single-slip system dominated, with a large number of dislocations cutting into the γ′ phase becoming the leading factor in the microscopic deformation mechanism.

The literature explains that lattice rotation and slip deformation are the primary mechanisms of deformation in single-crystal superalloys [24,30]. This study observes significant differences in lattice rotation and microstructural deformation organization among specimens with different deviation angles. The innovation of this paper lies in the interpretation of the differences in the degree of involvement of intragranular lattice rotation and slip systems to elucidate the mechanisms governing the varied mechanical properties and deformation behaviors of specimens at different angles from the [001] direction.

### 4.1. Lattice Rotation

When single-crystal superalloys are subjected to asymmetric loads due to the deviation between the specimen axis and the [001] crystal axis, lattice rotation occurs, with the direction of rotation aligning with the slip direction. During this process, the crystal coordinate system changes continuously, leading to changes in orientation after tensile deformation. Once the lattice rotates to a certain extent, slip systems are activated, completing the subsequent deformation and fracture processes. Samples with larger deviation angles exhibit higher elongation and cross-sectional shrinkage after tensile testing, and a higher degree of macroscopic elliptical extent of the fracture, indicating significant lattice rotation and substantial changes in crystal orientation after rotation. The primary slip system of single-crystal superalloys is {111}<110> octahedral slip, supplemented by {111}<112> hexahedral secondary slip systems. Previous literature has predominantly investigated lattice rotation during creep deformation processes in single-crystal superalloys [31,32]. The deformation mechanism during creep is characterized by {111}<112> activation, which differs significantly from the deformation mechanism at room temperature involving {111}<110>. Additionally, variations exist in the direction and path of lattice rotation.

**Figure 10:**
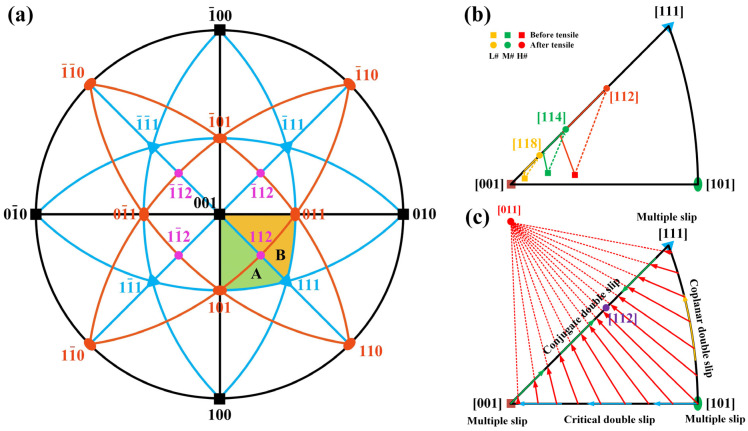
(**a**) The standard [001] stereographic projection. (**b**) The lattice rotation path of different misorientations during RT tensile deformation. (**c**) Illustration of lattice rotation in a standard stereographic triangle under {111} <110> slip.

Figure 10 illustrates the lattice rotation process under the {111}<110> slip system, using a standard stereographic triangle. Figure 10c shows that within the [001]–[101]–[111] standard stereographic triangle, multiple slip systems are activated at the [001], [101], and [111] poles, with the [001]–[101], [001]–[111], and [101]–[111] boundaries being areas of double slip, whereas the interior region of the triangle activates only one slip system [23]. Although all three boundaries belong to double-slip systems, they correspond to different slip characteristics: [001]-[101] as critical double slip, [101]–[111] as coplanar double slip, and [001]–[111] as conjugate double slip [33,34,35,36,37]. For samples oriented within the triangle, only the (11$\bar{1}$) [011] slip system is activated, causing rotation towards the [011] slip direction. Upon rotation to the [001]–[111] boundary, the sample is at the intersection of two symmetric stereographic triangles, as shown in areas labeled A and B in Figure 10a. Thus, the ($\bar{1}$11) [101] slip system is activated in [001]–[011]–[111] (label B), followed by lattice rotation along the [001]–[111] boundary under the influence of conjugate double slip until the [112] position reached, where the sample no longer rotates due to the counteracting effects of two slip systems [32]. Figure 10b displays the orientation changes in samples with different deviation angles measured before and after the experiment using SC-XRD and EBSD, respectively. The L#, M#, and H# samples rotated from their initial positions within the triangle, deviating approximately 3°, 8°, and 13° from the [001] direction, towards near [118], near [114], and near [112] orientations along the [001]–[111] boundary. According to the theoretical rotation path shown in Figure 10c, each sample first rotates from inside the triangle to the [001]–[111] boundary and then rotates along the boundary to the [118], [114], and [112] orientations, respectively. The length of the lattice rotation path increases with the initial deviation angle, which is the main reason for the differences in elongation, cross-sectional shrinkage, and the degree of macroscopic elliptical extent of the fractures among samples with different deviation angles.

### 4.2. Deformation Mechanism

Single-crystal superalloys, as γ/γ′ phase alloys, have their mechanical properties and deformation behaviors significantly influenced by the manner in which dislocations traverse the γ′ precipitate phase. The primary modes of dislocation interaction with the γ′ precipitate phase include: (i) paired dislocations shearing the γ′ phase, involving the antiphase boundary (APB); (ii) shearing of the γ′ phase by superlattice stacking faults (SSFs) and SSF loops; (iii) dislocations bypassing the γ′ phase through the Orowan mechanism; (iv) dislocations climbing over the γ′ phase under thermal activation [38]. Different deformation mechanisms dominate under various experimental conditions, such as temperature, and at different stages of deformation [39,40]. These mechanisms compete and interact with each other.

After tensile deformation, samples with a lower deviation angle (L# orientation) exhibit low dislocation density within the γ channels and visible SSFs. In previous studies, SSFs within the γ channels have been observed. During tensile deformation, it is generally believed that a/2<101> interface dislocation decomposes at the γ/γ′ interface, with two a/6<112> Shockley dislocations continuously separating under the drive of flow stress and misfit stress between γ and γ′, thereby generating stacking faults in the γ matrix. This generation process is described by Formula (2) [41]:

a/2<101>→a/6<112>+a/6<121>+SSF in γ matrix(2)

Furthermore, after the decomposition of interface dislocations a/2<101>, a/3<121> superlattice intrinsic stacking faults shear into the γ′ phase, resulting in stacking faults within the γ′ phase, and leaving behind a/6<112> Shockley dislocations at the γ/γ′ interface [42]. The detailed reaction equation is illustrated in Formula (3):

a/2<101>→a/6<112>+a/3<121>+SSF in γ′ phase(3)

The partial dislocation a/3<121> can shear into the γ′ phase, while the Shockley dislocation a/6<112> finds it difficult to do so, resulting in a significant accumulation of sessile dislocations within the matrix. For the orientation L# with a deviation angle of 3°, stacking faults occur in two directions. Literature indicates that stacking faults are less common in single-crystal superalloys at room temperature, predominantly occurring in a single direction [41,43,44]. However, specimens with the L# orientation exhibit stacking faults in two directions, possibly due to a lower stacking fault energy (SFE), indicating slip in two different directions. The interaction and constraint between these stacking faults are the primary reasons for work hardening, leading to higher strength, but lower elongation and reduction in area. Additionally, SSF loops are observed in the γ′ phase with orientation L#, formed in the manner described by Formula (4) [45]. Due to the relatively low thermal activation and mismatch stress at room temperature, the number of SSF loops is significantly less than that of SSFs.

a/2<101>→a/3<112>+a/3<121>+SSF loop in γ′ phase(4)

For samples oriented at an 8° deviation angle (M# orientation), SSFs in the γ′ phase predominantly occur in one direction, indicating the primary activation of a single-slip system, leading to reduced work hardening, decreased strength, and increased elongation and cross-sectional shrinkage. Simultaneously, the dislocation density inside the γ channels increases significantly, making SSFs difficult to identify, but a considerable accumulation of Shockley partial dislocations a/6<112> is observed, resulting from entanglements of dislocations generated during lattice rotation. Short, straight dislocations appear in the γ′ phase, mainly the a/3<121> partial dislocations shearing into the γ′ phase, along with a few curved dislocations. For samples oriented at a 13° deviation angle (H# orientation), compared to the M# orientation, the entanglement level of dislocations in the γ channels further increases, suggesting that the increased degree of lattice rotation is accompanied by a significant proliferation and entanglement of the a/6<112> Shockley partial dislocations. A large number of partial dislocations appear in the γ′ phase, with fewer SSFs and primarily in a single direction, indicating the dominance of a single-slip system.

In summary, lattice rotation and crystal slip dominated by {111}<110> octahedral slip are the primary mechanisms of room temperature tensile deformation in single-crystal superalloys. The lattice rotation mechanism involves an initial rotation within the standard triangle to the [001]–[111] boundary, followed by rotation towards the [112] orientation along this boundary. The crystal-slip micro-mechanism is characterized by the shearing of SSFs through the γ′ phase. The different involvement of these two mechanisms in samples with varying deviation angles is the main cause of the differences in tensile performance and deformation behavior. At a 3° deviation angle, the small difference between the tensile and crystal axes, short path of lattice rotation, and the low degree of rotation lead to low ductility, with crystal slip being the dominant deformation mechanism during stretching. The mutual constraint of SSFs in two directions on the {111} plane in the γ′ phase leads to work hardening, which is the main reason for the increased strength. As the deviation angle increases, under the influence of asymmetric loading, the path of lattice rotation lengthens, and the contribution of deformation caused by lattice rotation becomes more significant, leading to a gradual increase in ductility and fracture ellipticity in tensile deformation, accompanied by a significant accumulation of Shockley partial dislocations a/6<112> within the γ channels. Fracture occurs when lattice rotation reaches an orientation that activates a slip system, with SSFs in a single direction within the γ′ phase leading the process, resulting in reduced work hardening and strength.

This paper provides new insights and research directions on the performance variations and corresponding deformation mechanisms caused by small-angle deviations from the [001] direction in single-crystal superalloys. Based on this, there is much more content to explore. On one hand, the deformation mechanism of single-crystal superalloys is highly dependent on temperature changes, making it necessary to conduct mechanical tests and studies related to deviation angles near the [001] direction at different temperatures. On the other hand, as the development of single-crystal superalloy compositions progresses, with an increase in the content of refractory elements like Re and Ru, changes will occur in the γ/γ′ misfit and stacking fault energy, affecting the generation and movement of micro-dislocations. Therefore, conducting research based on deviation angles near the [001] direction for different alloy compositions will be of guiding significance for the research and development and engineering applications of materials.

## 5. Conclusions

The study analyzed the room-temperature tensile properties of single-crystal superalloy samples near the [001] orientation at various deviation angles and investigated the mechanisms of deformation features at different angles. The reasons for the differences in mechanical properties and damage behavior were explained in terms of lattice rotation and crystal slip. The results can be summarized as follows:

(1) Within the vicinity of the [001] direction in single-crystal superalloys, samples oriented at deviation angles of 3°, 8°, and 13° exhibited decreasing strength and increasing ductility with increasing deviation angle.
(2) Lattice rotation and crystal slip, dominated by {111}<110> octahedral slip, are the primary mechanisms of room-temperature tensile deformation in single-crystal superalloys. The varying degrees of involvement of these two mechanisms in samples at different deviation angles are the main reasons for the differences in tensile performance and deformation behavior.
(3) At a 3° low misorientation angle, the short path and low degree of lattice rotation, along with the mutual constraint of the SSFs in two directions on the {111} plane in the γ′ phase leading to work hardening, result in low ductility and high strength at room temperature. As the misorientation angle increases, the lengthening of the lattice rotation path and the significant accumulation of Shockley partial dislocations a/6<112> within the γ channels, with SSFs in a single direction dominating within the γ′ phase, are the main reasons for the increased ductility and reduced strength.
(4) Drawing on the insights from this study, further investigations should examine how temperature fluctuations and misorientation angles affect deformation mechanisms, as well as the role of refractory elements like Re and Ru in altering the microstructural features (e.g., γ/γ′ misfit and stacking fault energy) and deformation responses of single-crystal superalloys. Such research is vital for refining alloy compositions and boosting their applicational efficacy.

## Figures and Tables

**Table 1:** The chemical composition of the nickel-based single-crystal superalloy (wt.%).

Cr	Co	W	Mo	Al	Ta	Nb	Re	Hf	C	Ni
4.2	8.8	8.0	2.0	5.6	7.2	0.5	1.8	1.0	0.012	Bal

**Table 2:** Retest results of single-crystal superalloy tensile specimens misorientation.

Casting Ingots Number	Specimen Number	Misorientation/°			Standard Stereographic Triangle
		[001]	[011]	[111]	
L#	1#	3.1	42.4	51.5	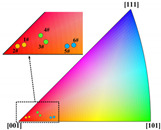
	2#	2.1	43.0	52.8	
M#	3#	7.5	37.8	48.2	
	4#	6.8	40.1	47.9	
H#	5#	11.1	33.9	46.6	
	6#	12.0	33.0	45.9	

**Table 3:** Tensile properties of single-crystal superalloy with different misorientations.

Casting Ingots Number	Specimen Number	Deviation from [001]	Ultimate Tensile Strength/MPa	Yield Strength/MPa	Elongation/%	Cross-Sectional Shrinkage/%
L#	1#	3.1	1100	996	10.8	16.0
	2#	2.1	1112	1024	14.3	16.0
M#	3#	7.5	998	959	17.3	19.6
	4#	6.8	1032	982	16.5	18.6
H#	5#	11.1	927	901	31.5	25.0
	6#	12.0	926	895	35.6	27.6

## Data Availability

Data are contained within the article.
